# How to become a Multinational Association of Supportive Care in Cancer-designated center of excellence in supportive care in cancer

**DOI:** 10.1097/CCO.0000000000000949

**Published:** 2023-04-27

**Authors:** Andrea Antonuzzo, Maurizio Lucchesi, Carla Ida Ripamonti

**Affiliations:** a University Hospital of Pisa, Pisa; b Department of Medical Oncology – Azienda USL Toscana Nord Ovest; c University of Brescia, Brescia, Italy

**Keywords:** designated centers of excellence, Multinational Association of Supportive Care in Cancer, supportive care

## Abstract

**Purpose of review:**

Aim of this review is to encourage and involve more doctors to take care of supportive care in cancer patients and to become centers of excellence.

**Recent findings:**

In 2019, MASCC initiated a certification program to recognize oncology centers that demonstrate best practices in supportive cancer care but literature on how to become MASCC-designated center of Excellence in Supportive Care in Cancer is scarce and will be bulleted.

**Summary:**

Becoming centers of excellence means not only the recognition of the clinical and managerial requirements to provide good supportive care but also the creation of a network of centers to participate in multicenter scientific projects and thus improve knowledge in the field of supportive care in cancer patients.

## INTRODUCTION

According to the Multinational Association of Supportive Care in Cancer (MASCC), Supportive care is the prevention and management of the adverse effects of cancer and its treatment. This includes management of physical and psychological symptoms and side effects across the continuum of the cancer journey from diagnosis through treatment to posttreatment care. Supportive care aims to improve quality of life during anticancer treatments, quality of rehabilitation, secondary cancer prevention, survivorship, and end-of-life care [1]. 

**Box 1:**
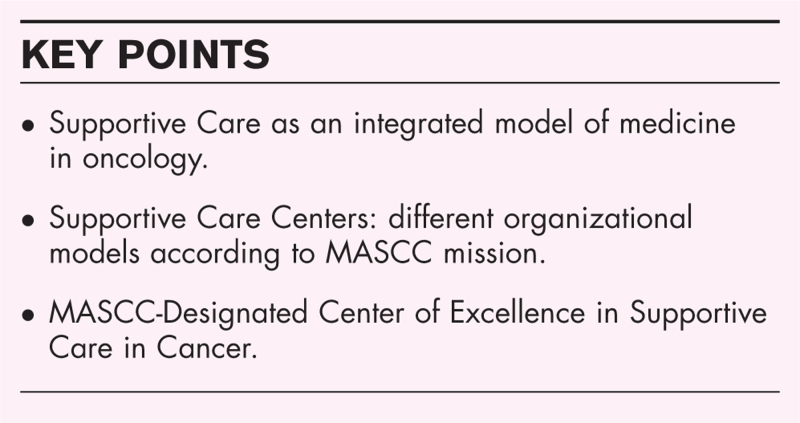
no caption available

Milestones in supportive care in cancer:

(1) Supportive care aims to maintain (or improve) quality of life, and to ensure that people with cancer can achieve maximum benefit from their anticancer treatment.
(2) Supportive care is relevant throughout the continuum of the cancer experience from diagnosis through treatment to posttreatment care (and encompasses cancer survivorship, and palliative and end-of-life care).
(3) Supportive care involves a coordinated, person-centric, holistic (whole-person) approach, which should be guided by the individual's preferences, and should include appropriate support of their family and friends.
(4) Supportive care is a basic right for all people with cancer, irrespective of their personal circumstances, their type of cancer, their stage of cancer, or their anticancer treatment. It should be available in all cancer centers, and other medical facilities that routinely manage people with cancer.
(5) Supportive care represents an integrated model of medicine in oncology that in respect to other integration models fills a phase of the disease, which is related to oncologic treatments, clinical trials to allow cancer therapy to reach the effective dose-intensity and dosing interval thus allowing a global well being of the patients.

Supportive care is based on a multidisciplinary and multispecialistic approach.

## SUPPORTIVE CARE CENTERS

Supportive care centers represent an alternative reference to emergency room and hospitalization for patients who are in adjuvant or palliative cancer treatments and require supportive medical pharmacological therapies aimed to monitor and treat the related toxicity thus to ensure greater adherence to the treatment protocols in terms of dose-intensity and interval of administration [2–4].

## DESIGNATED CENTERS OF EXCELLENCE IN SUPPORTIVE CARE IN CANCER

In 2019, MASCC initiated a certification program to recognize oncology centers that demonstrate best practices in supportive cancer care. Such centers are designated by MASCC as Centers of Excellence in Supportive Care in Cancer. Through this initiative, MASCC promotes and recognizes cancer care centers around the world that are successfully integrating oncology and supportive services. Such centers are recognized by MASCC for upholding high standards and providing comprehensive services in supportive cancer care. MASCC's certification program aims both to educate and to encourage a supportive care focus among oncology healthcare professionals [1].

Any cancer center or department may apply for MASCC certification, and there is no fee to do so. Applications are reviewed by MASCC's Certification Program Committee, which in turn makes recommendations to MASCC's Executive Committee for final decisions. Certification criteria include a supportive care focus in clinical activities, research, and educational initiatives, as well as adherence to international guidelines [1].

Aims of MASCC's certification program are

(1) to promote and recognize oncology centers around the world that demonstrate best practices in supportive cancer care by successfully integrating oncology and supportive care, upholding high standards, and maintaining comprehensive supportive care services.
(2) to educate and to encourage a supportive care focus among oncology, internal medicine, geriatricians, healthcare professionals globally.
(3) to recognize oncology centers for upholding high standards and providing comprehensive services in supportive cancer care, to recognize centers around the world that are successfully integrating oncology and supportive services.

### Who should be dedicated to supportive care?

MASCC certification provides for the application of a physician or nurse directly involved in supportive care, regardless of specialization but who knows medical oncology, the adverse effects of therapies, their prevention, and their treatment.

Although supportive care is closely linked to medical oncology and radio-oncology, it has no specific specialty. However, because of the type of disease, adverse effects, and comorbidities, the presence of at least a medical oncologist, an internist, and a geriatric is the basis for a much broader multidisciplinary collaboration. In fact, most of the toxicities are related to internal medicine and geriatrics and may require diagnostic tests and pharmacological intravenous therapies.

### How to apply?

**FIGURE 1:**
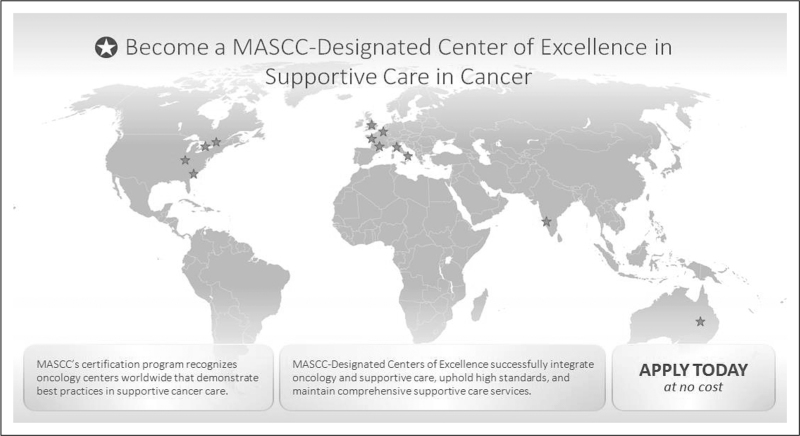
The stars in the picture represent geographic areas of the 20 centers, mostly in Europe, actually certificated as MASCC-designated Center of Excellence in Supportive Care in Cancer.

The first step to become a MASCC-designated Center of Excellence in Supportive Care in Cancer is downloading the application form from the MASCC website [1], complete it in its entirety and also write a narrative part that tells how supportive care was born in the structure to which the applicant belongs, what are the daily activities that take place from the infusion therapies, relational point of view with the patient and family. Moreover, MASCC wants to know the basis of multidisciplinary and multispecialty, the collaboration with palliative care specialists and how the center has evolved since its establishment, where is it located (in the Oncological, Radiotherapy Department, together with Palliative Care, etc.). Main aims and benefits to become a MASCC Center of Excellence in supportive care in cancer are summarized in Fig. 1.

## MULTINATIONAL ASSOCIATION OF SUPPORTIVE CARE IN CANCER EVALUATION CRITERIA AND FORM

MASCC evaluation criteria are:

(1) Centrality of patient
(2) Dedicated organizational models
(3) Dedicated activity
(4) Multidisciplinary staff
(5) Teaching programs
(6) Adherence to guidelines

Certification criteria include five parts:

(1) general,
(2) a supportive care focus in clinical activities,
(3) logistics,
(4) research, and educational initiatives,
(5) adherence to international guidelines.

Each part, excluding general, has a score. The scoring system has been realized between 0 and 100 to better synthesize with a number the center engagement on supportive care in cancer patients (0–100 is the most intuitive scale).

The minimum score that allows the definition of MASCC-Designated Centers of Excellence in Supportive Care in Cancer is 60 [5^▪^].

### Part 1: general

General information on the center and the applicant. This information will be compared with the other parts of the form. Any type of incoherence will generate a query to the applicant.

MASCC wants to know:

(1) what type of institute is related to the application?
(2) what types of cancer are treated (solid, hematologic, both)?
(3) what patient age group (pediatric, adult, geriatric)?
(4) services available: medical oncology, radiation oncology, hematology, supportive care, palliative care (for each service the availability of inpatients, day care, outpatients),
(5) availability of emergency care for treatment-related toxicities.

### Part 2: a focus on clinical activities

This is the core of the evaluation. This part of the score evaluates how the center organizes the clinical activities around the concept of supportive care, trying to catch the heterogeneous solutions through a descriptive detailed text and specific questions.

MASCC wants to know:

(1) which supportive care therapies are provided to patients receiving cancer treatment at the center and in which setting are the treatment provided. Detailed information is required about the settings for each supportive therapy.
(2) how are out-patients with treatment-related toxicity managed?
(3) who are the people involved in the core supportive care team?

This part needs particular attention regarding:

(a) Main reasons for requiring a visit: uncontrolled symptoms, toxicities, and patient's logistic issues.
(b) Physical examination, blood tests, first-level radiological examinations.
(c) Direct management of clinical cases together with other specialists (e.g. nutrition, pain, anesthesiology, dermatology, endocrinology, cardiology).
(d) Daily planned or not activity given by chaplain and psychologist for the spiritual and psychological needs of the patients.

(4) does the service use validate symptom and quality of life assessment tools in the routine clinical practice?
(5) does the service provide nonpharmacological support?
(6) another information regards palliative care features at the center of applicant.
(7) which are the types and level of integration between supportive care and palliative care staff?
(8) how is end-of-life care and symptom management provided?

### Part 3: logistic

(1) in what settings is supportive care provided: inside or outside-dedicated structures?
(2) how many hours per day and how many days per week is care provided?
(3) is care available for unplanned patient’ visits (as outpatients or day service)?
(4) does the service provide dedicated contacts with patients? How long during the day? By what means (telephone, emails, fax line, etc.)?

### Part 4: research and educational initiatives

MASCC wants to evaluate:

(1) in what types of research are center staff involved in oncology, supportive care in cancer patients, palliative care in cancer patients?
(2) the research is basic, translational, clinical?
(3) who are the professions of the center's researchers?
(4) are there SC publications in peer-reviewed journals?
(5) which type of educational activities are organized in the center?

### Part 5: adherence to international guidelines

Adherence to International Guidelines is an important part for MASCC evaluation.

## THE FIRST CENTERS OF EXCELLENCE

In 2020, the first *MASCC-designated Center of Excellence in Supportive Care in Cancer* was the Dedicated Supportive Care Unit at the Fondazione IRCCS, Istituto Nazionale dei Tumori (INT) di Milano. This Center born in June 2009, was considered as a new integrated model of medicine in oncology [2]. The main aims of the Italian Center are: collaboration with all the Units of the INT and with all the specialists to work in a multidisciplinary setting; provide a medical therapy to support the patient, from diagnosis and throughout the oncological treatment (adjuvant/palliative), for the treatment of adverse events, toxicity and comorbidities, in order to ensure the patient's psychophysical well being and improve adherence to treatment protocols in terms of dose-intensity and administration interval; follow national (AIOM Associazione Italiana di Oncologia Medica) and international guidelines (European Society of Medical Oncology – ESMO, American Society of Clinical Oncology – ASCO; MASCC); evaluate the needs related to information, communication, expression of emotions, social, spiritual/existential and financial assistance, as well as the need to search for meaning regarding the new dimension of life (phenomenological position of Victor Frankl), to ensure that the person is taken in charge in its entirety; provide support to family, survivors and health personnel involved in day-to-day care; promote pharmacological and nonpharmacological research; promote teaching activities.

All the activities are now performed by five physicians (two oncologists, two internists, one geriatrician), four RNs, three healthcare workers, and four volunteers selected and supervised by the Italian League Against Cancer (LILT).

The second *MASCC-designated Center of Excellence in Supportive Care in Cancer* is inside Pisa Medical Oncology (a Department of the University Hospital) from 2012, with a new model of working based on the direct assistance to patients with a new ambulatory room inside the oncological day hospital dedicated to symptoms and toxicities evaluation and management [3,4,6,7]. The activity is conducted by one dedicated oncologist and two nurses together with two postgraduate medical doctors per day.

Supportive Care Service works 6 days per week in the morning for planned and unplanned adult patients with solid tumors (types of cancer and age) who enter the oncology department because of complications from treatment and gives an emergency telephone service through a mobile line for clinical enquiries. In the department are available beds for out and inpatients in oncology, radiotherapy, and hematology wards.

The early recognition of clinical problems may consent to treat them in the same day of access and in the next days if necessary, avoiding Emergency Room access in the same hospital. Symptoms and toxicities are evaluated also with the use of PROs (Patient Reported Outcomes) that better describe the patient's health condition, significantly reduce admission to Emergency Room and to Hospital and improve QoL of the patients [8,9^▪^].

The administration of symptomatic therapy (e.g. intravenous fluid infusions, collecting of blood samples and radiographic examination, pain, and nutritional therapy) and other specialists’ further evaluations are feasible and direct. All the clinical activity is based on scientific society guidelines (MASCC, ESMO, ASCO, AIOM).

Moreover, everyday chaplain and psychologist may be involved in the clinical assistance for patient's spiritual and psychological needs. The psychologist, when necessary, in case of urgent consultation, cooperates with psychiatric ward. At the same time, there are an active program of palliative care consultation to provide adequate assistance for patients with advanced and not more treatable disease (according to ESMO, palliative care guidelines). This kind of activity is performed in collaboration with healthcare professional involved in homecare services and together with general practitioners.

The majority of visited outpatients have metastatic disease and are receiving active anticancer treatment. The main reasons for requiring a visit are uncontrolled symptoms and toxicities (pain, fatigue, anorexia, fever, diarrhea, nausea/vomiting). Only a small part of them require admission in our inpatients ward or to their wards through ER.

Across the years until today, this activity does increase and maintain its number of assisted patients (more than 1000 per year).

Positive and direct effect because of the SCC is a reduction by 3,2% of the number of unplanned hospitalizations of on-treatment cancer patients and the consequent net reduction by 2,2% of the costs used for unplanned hospitalizations. Furthermore, a reduction of about 5% in the ER accesses [5^▪^] was described. Another important clinical and economic effect, is the marked decrease in the costs associated with red blood cell transfusions (net reduction of one-third of the total expense) [6], deeply according to the results of a previous study by Ripamonti *et al.*[10].

From the beginning, Supportive Care Service, does organize weekly staff rounds to discuss clinical cases and periodical meetings with the department healthcare professionals to widespread supportive care knowledge.

It is also maintained an active participation in national collaborative research studies in the field of supportive care and, furthermore, writing of paper for peer reviewed journals and abstracts submitted to national or international meetings.

In 2019, a narrative review by Northfield *et al.*[11] enhances both rationale and results of our models of care considering them essential for better patient's outcomes.

The opportunity to create these services in different oncological contests may be an interesting opportunity for all healthcare providers. Until December 2022, MASCC have designated 20 Center of Excellence in Supportive Care in Cancer worldwide, mostly in Europe. The excellence in supportive care has been documented in different type of structures, involving the cure of pediatric, adult, or geriatric cancer patients. Denomination and location of these centers are summarized in Table 1 and Fig. 1.

**Table 1:** MASCC-designated Center of Excellence in Supportive Care in Cancer 2019–2022

#	Center	Location
1	Département d’Oncologie Médicale et Soins de Support Hôpital Foch	Suresnes, France
2	Division of Cancer Services, Metro South Health – Princess Alexandra Hospital	Queensland, Australia
3	Gustave Roussy Cancer Campus, Interdisciplinary Cancer Course Department (DIOPP)	Paris, France
4	Institut Jules Bordet	Brussels, Belgium
5	Levine Cancer Institute – Atrium Heath, Department of Supportive Oncology	Charlotte, NC, USA
6	Fondazione IRCCS, Istituto Nazionale Tumori Milano: Oncologia – Cure di Supporto al paziente	Milano, Italy
7	Terapie di supporto al paziente oncologico – Azienda Ospedaliero-Universitaria Pisana	Pisa, Italy
8	Leeds Children's Hospital	Leeds, UK
9	The Ohio State University Comprehensive Cancer Center – James Cancer Hospital and Solove Research Institute	Columbus, Ohio, USA
10	The Royal Surrey NHS Foundation Trust	Guilford, UK
11	The Clatterbridge Cancer Centre NHS Foundation Trust	Liverpool, UK
12	Fondazione IRCCS Istituto Nazionale Tumori: Pediatric Unit	Milano, Italy
13	Manipal Comprehensive Cancer Care Centre, KMC	Manipal, India
14	G. Mazzini Hospital Medical Oncology Unit	Teramo, Italy
15	Azienda Ospedaliero Universitaria Sant’Andrea	Rome, Italy
16	Southampton Children's Hospital - Department of Paediatric Oncology and Haematology	Southampton, UK
17	Children's Hospital London Health Sciences Centre, Division of Hematology/Oncology	London, Canada
18	The Hospital for Sick Children (SickKids), Garron Family Cancer Centre	Toronto, Canada
19	Children's Hospital of Eastern Ontario, Division of Hematology/Oncology	Ottawa, Canada
20	McMaster Children's Hospital, Hamilton Health Sciences, Division of Hematology and Oncology	Hamilton, Canada

Again, the further way to increase the quality of these services through the MASCC certification as a center of excellence, is another effort to have better cancer care.

## CONCLUSION

MASCC certification as a Center of Excellence in Supportive Care in Cancer includes the following benefits:

(1) Use of the MASCC logo on marketing and advertising related to the center or program.
(2) Use of the title ‘A MASCC-Designated Center of Excellence in Supportive Care in Cancer’.

Applications are accepted on a rolling basis. Certification is valid for 3 years, after which the center must apply for re-evaluation. Centers with which members of the Certification Program Committee are affiliated may apply. However, the relevant committee members must recuse themselves from the evaluation process. Candidates are encouraged to become MASCC members and promote the membership in their institution.

## Acknowledgements

*We would like to thank the Multinational Association Supportive Care in Cancer (MASCC).*

### Financial support and sponsorship

*None.*

### Conflicts of interest

*There are no conflicts of interest.*

## REFERENCES AND RECOMMENDED READING

Papers of particular interest, published within the annual period of review, have been highlighted as:

▪ of special interest
▪▪ of outstanding interest
